# The nucleotide exchange factor, GrpE, modulates substrate affinity by interaction of its N-terminal tails with the DnaK substrate–binding domain

**DOI:** 10.1016/j.jbc.2026.111176

**Published:** 2026-01-20

**Authors:** Akshitha Maqtedar, Maria-Agustina Rossi, Eugenia M. Clerico, Robert V. Williams, Lila M. Gierasch

**Affiliations:** 1 Department of Biochemistry & Molecular Biology, University of Massachusetts Amherst, Amherst, Massachusetts, USA; 2 Department of Chemistry, University of Massachusetts Amherst, Amherst, Massachusetts, USA

**Keywords:** 70-kDa heat shock protein, chaperone, GrpE, DnaK, NMR, nucleotide exchange factors

## Abstract

The 70-kDa heat shock proteins assist in protein folding through allosteric communication between their nucleotide-binding domains and substrate-binding domains (SBDs), which are connected by an interdomain linker. Their nucleotide-dependent allosteric cycle is modulated by ligand binding and cochaperones, including nucleotide exchange factors. GrpE, the nucleotide exchange factor for the *Escherichia coli* 70-kDa heat shock protein, DnaK, has been proposed to have a dual effect on the chaperone, facilitating the exchange of ADP for ATP in the nucleotide-binding domain in a temperature-dependent fashion and promoting substrate release from the SBD. We recently reported NMR-based evidence that GrpE binding to DnaK has a direct structural effect on the SBD. Here, we built on these findings and provide new evidence supporting a model in which the disordered N-terminal tails of GrpE facilitate peptide dissociation from the nucleotide-free DnaK–GrpE complex by transiently binding to the canonical substrate-binding site in the SBD. This GrpE–SBD interaction, while weak, is favored by the high local concentration of the tails around the SBD after complex formation and provides a direct mechanism to facilitate substrate release in addition to the potential more indirect allosteric mechanism arising from a GrpE–SBD conformational shift. Moreover, we identified the DnaK binding motif in GrpE’s N-terminal disordered tails as ^17^IIM^19^, which is highly conserved in bacteria. Excitingly, our data further suggest a mechanism for the temperature dependence of GrpE’s modulation of DnaK’s refolding activity: as the temperature increases, unfolding of GrpE’s coiled-coil weakens its contacts with the SBD, reducing N-terminal tail binding, thus increasing the affinity of DnaK to substrates.

Molecular chaperones play key roles in maintaining cellular protein health in all organisms (1). Central players among molecular chaperones are the 70-kDa heat shock proteins (Hsp70s) (2, 3). The structure of Hsp70s is conserved across prokaryotes and eukaryotes and consists of two domains—an N-terminal 44-kDa nucleotide-binding domain (NBD) and a C-terminal 30-kDa substrate-binding domain (SBD)—connected by a conserved interdomain linker. Hsp70s function *via* a nucleotide-dependent allosteric cycle based on a transition between two conformations, ATP and ADP bound, modulated by binding to incompletely folded substrate proteins and interacting with cochaperones. One family of cochaperones comprises the nucleotide exchange factors (NEFs) that facilitate the exchange of ADP for ATP in the NBD of all Hsp70s (3, 4). While the major role of NEFs is nucleotide exchange, it has also been observed that NEFs promote substrate release from the Hsp70 SBD. This functional role arises in part from NEF facilitation of the transition of Hsp70s from the ADP-bound high substrate affinity state to the ATP-bound low substrate affinity state but may also be a consequence of a direct action of the NEF on the Hsp70 SBD (5, 6, 7, 8, 9).

Hsp70 NEFs are categorized into four classes: BAG proteins, HspBP1 and Hsp110 in eukaryotes, and GrpE in prokaryotes, mitochondria, and chloroplasts (3, 10). GrpE is the NEF of the *Escherichia coli* Hsp70 DnaK and is characterized by a distinctive structure and function. It is a homodimer with a unique cruciform shape consisting of a globular C-terminal region with two β-bundles and four α-helices, a long coiled-coil, and two N-terminal disordered tails (Fig. S1*A*) (7, 11). The coiled-coil, characterized by a melting transition with a *T*_m_ of 48 °C, confers upon GrpE the ability to act as a cellular thermosensor (12, 13). At 37 °C and below, GrpE assists in the DnaK refolding activity of luciferase (14, 15). As the temperature increases, the GrpE coiled-coil domain starts to unfold, and GrpE loses its ability to assist DnaK in protein refolding (13, 16).

GrpE appears to exploit the dual role described above for NEFs in general in its facilitation of the role of DnaK in protein refolding. First, GrpE accelerates the Hsp70 allosteric cycle *via* its well-established promotion of the exchange of ADP by ATP. For this role, GrpE binds to the ADP-bound NBD and increases the nucleotide off rate by stabilizing subdomain IIB in lobe II in a conformation that opens the nucleotide-binding cleft (4, 10). In this mechanism, it is important to note, as we reported previously (17), that the ADP-bound NBD–GrpE complex is of low stability and has increased conformational heterogeneity in comparison to the stable nucleotide-free NBD–GrpE complex. Thus, GrpE promotes the release of ADP, poising the NBD for ATP binding, and facilitating the resulting conformational shift of DnaK to a low substrate affinity state. The second role of GrpE has been suggested by several studies that report weakening of the binding affinity of substrate to DnaK upon binding to GrpE (5, 6, 7). In an early structural study of the GrpE complex with DnaK, Harrison *et al.* (7) reported that full-length GrpE weakened the binding of DnaK to an unfolded polypeptide substrate, but that N-terminally truncated GrpE did not. Follow-up work has explored how the kinetics of substrate binding to DnaK are affected by the binding of GrpE (5, 6, 9) and the necessity for the 33-residue N-terminal mobile region to be present for the enhancement of substrate release (5, 6, 7, 9).

In terms of a mechanism for the impact of GrpE binding on DnaK substrate affinity, previous studies raised the possibility that the 33-amino acid N-terminal disordered tails interact with the canonical substrate-binding site in the DnaK SBD to assist in peptide release (5, 6, 7, 8, 9), but no direct interaction has been reported thus far. Removal of the N-terminal 33 amino acids (in a construct called GrpE^33–197^) abrogated the impact of GrpE complex formation on substrate binding (7, 9). Notably, the previous studies examining these effects of DnaK–GrpE complex formation on substrate affinity focused on ADP-bound DnaK, which our previous study suggested is not the favored species in the ensemble of GrpE-bound DnaK chaperones. In previous and current studies, we have examined the complex of GrpE and nucleotide-free DnaK, given that our observations supported the greater stability and conformational homogeneity of this state.

In our previous study (17), we also reported NMR chemical shift perturbations (CSPs) in the DnaK SBD upon GrpE binding to DnaK, supporting previous biochemical findings of GrpE-induced alteration of substrate affinity (5, 6, 7, 9). Two possible mechanisms for the impact of GrpE binding on the SBD were suggested: (1) transmission of an allosteric signal from the NBD to the interdomain linker, and then to the SBD or (2) a direct interaction between the GrpE N-terminal disordered tails and the DnaK SBD (17).

There are now three atomic resolution structures of DnaK–GrpE complexes from *E. coli* DnaK homologs (8, 18, 19) that provide structural insights into the nature of the interaction between *E. coli* GrpE and the DnaK SBD: the cryo-EM structure of the human mortalin–GrpEL1 complex (Fig. S1*C*) (18), the X-ray crystal structure of the *Geobacillus kaustophilus* DnaK–GrpE (Fig. S1*D*) (19), and the cryo-EM structure of the *Mycobacterium tuberculosis* DnaK–GrpE complex (Fig. S1*B*) (8). These structures reveal three significant contact regions between the NEF and Hsp70, which are designated I to III using descriptors from the structure of the *M. tuberculosis* GrpE–DnaK complex (Fig. S1*B*) (8). Contact regions I and II involve interactions between the NEF and the NBD. Most exciting for the possible action of the NEF on substrate binding is the location of contact region III, wherein the DnaK SBD interacts with the coiled-coil domain of GrpE. Here, DnaK SBD loop R388–P393 (loop 1) interacts with the D68–R75 segment of the coiled-coil domain of GrpE, and DnaK residue D451 interacts with R64 from GrpE (*M. tuberculosis* numbering). In addition, the interdomain linker lies across the GrpE coiled-coil domain. Comparable interactions are observed, albeit with lower resolution, in the other two GrpE–DnaK structures of *E. coli* DnaK homologs (18, 19). While all these structures provide provocative information on the interaction of the coiled-coil region of GrpE with the DnaK interdomain linker and SBD, in none of them are the disordered N-terminal tails resolved, leaving their potential role in mediating substrate release unclear.

Here, we deploy a multidisciplinary approach to explore the impact of *E. coli* GrpE binding on the substrate affinity of DnaK. First, we use current powerful prediction methods to support the structural homology of the *E. coli* DnaK–GrpE complex with those of the homologs. Importantly, we find that key interaction regions in all the complexes are highly conserved across a broader set of DnaK–GrpE homologs, supporting the generality of the structure of the complex. We next explore in greater depth how the dynamic N-terminal tails of GrpE may participate in the mechanism of GrpE action on DnaK. We rely heavily on NMR spectroscopy, as it is well suited to studying highly dynamic regions and can shed light on the involvement of the GrpE N-terminal tails in the complex. On the basis of our findings, we propose a model in which the disordered N-terminal tails of GrpE actively facilitate peptide dissociation from the apo-DnaK–GrpE complex by competing with the exogenous substrate for binding to the SBD canonical substrate-binding site. Our data argue that the N-terminal disordered tails transiently bind to the SBD substrate-binding site with low affinity. Despite the low affinity of the N-terminal tail binding interaction, the covalent linkage of the tails to GrpE in the complex confers upon them a high local effective concentration. Furthermore, we have identified the DnaK binding motif in the GrpE N-terminal tail as ^17^IIM^19^, which is conserved across many bacterial species. Finally, our model provides an explanation for the reduced impact of DnaK–GrpE binding on substrate affinity and the loss of DnaK refolding activity at elevated temperatures (14, 15, 20). As temperature increases, the coiled-coil domain of GrpE begins to unfold (13, 16), weakening the GrpE contacts with the SBD, reducing the likelihood of the direct interaction of the N-terminal tails with the SBD and thus abrogating the GrpE effect on peptide release.

## Results

### Comparison of the predicted structure of the *E. coli* DnaK–GrpE complex and DnaK–GrpE complex from homologs supports structural conservation

**Figure 1:**
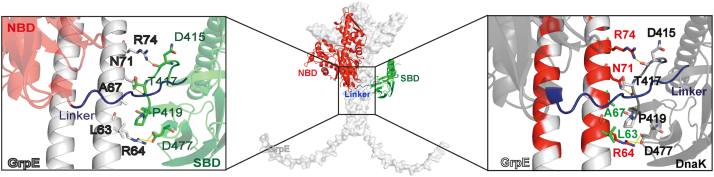
**AlphaFold2-predicted *Escherichia coli* DnaK–GrpE complex reveals conserved interaction modes**. The AlphaFold2-predicted structure of the *E. coli* DnaK–GrpE complex is shown in the center, and a close-up view of the interaction interface between DnaK SBD and GrpE coiled-coil domain is illustrated on the *right* and *left*. GrpE is highlighted in *white*, and in the Hsp70, the *NBD* is in *red*, the SBD is in *green*, and the interdomain linker is in *blue*. The *left panel* shows a zoomed view highlighting contacting residues between the GrpE coiled-coil (*light gray*) and DnaK SBD (*green*), whereas the *right panel* shows the same view with conserved GrpE residues indicated in *red*. DnaK, 70-kDa heat shock protein of prokaryote; GrpE, nucleotide exchange factor of prokaryote; Hsp70, 70-kDa heat shock protein; NBD, nucleotide-binding domain; SBD, substrate-binding domain.

As there has not been an experimentally determined structure for the complex of *E. coli* GrpE and full-length DnaK, we used AlphaFold2 to predict this complex (21, 22) (Fig. 1). As seen in the previously reported structures of complexes from homologous organisms, the NBD and SBD are not associated with one another in the *E. coli* DnaK–GrpE complex and instead adopt a conformation similar to the ADP-bound state of *E. coli* DnaK (23). The NBD–GrpE interactions are consistent with the previous X-ray structure of GrpE complexed with the isolated *E. coli* DnaK NBD (7) and the corresponding predicted structure from our laboratory (17). Excitingly, the contacts formed between the GrpE coiled-coil with DnaK are the same in the *E. coli* complex as in those of homologs (8, 18, 19) (Fig. S1, *B*, *C*, and *D*). These include interaction of the coiled-coil with SBD loop K414–P419 (loop 1), SBD residues N415, T417, D477, and the interdomain linker (*E. coli* numbering). The involvement of the interdomain linker in binding to the coiled-coil of GrpE is also consistent with previous biochemical observations showing a loss of proteolytic lability of this region in the DnaK–GrpE complex relative to its susceptibility in DnaK (6). Thus, three major interactions are implicated in the DnaK–GrpE complex: the DnaK NBD with the GrpE globular domain, the DnaK interdomain linker with the GrpE coiled-coil, and SBD loop 1 and residues N415, T417, and D477 with the GrpE coiled-coil.

**Figure 2:**
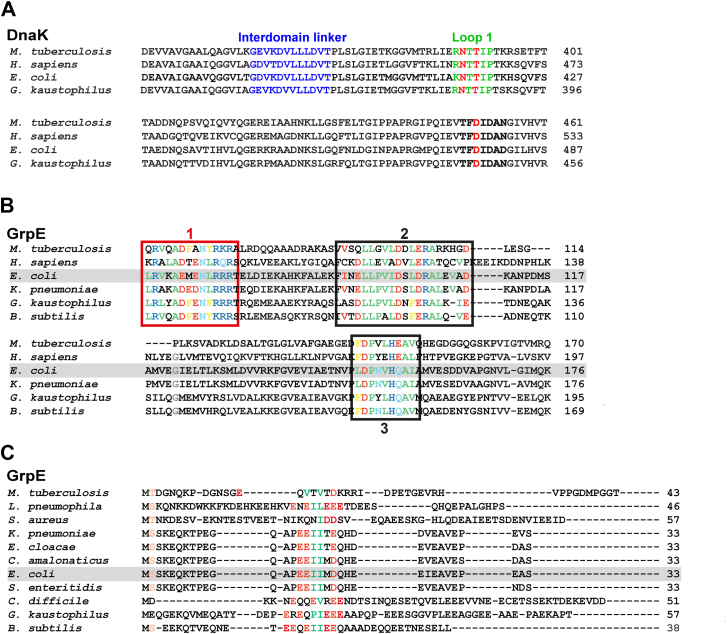
**DnaK and GrpE homologs exhibit sequence conservation in the interaction interfaces of the DnaK–GrpE complex**. *A,* sequence alignment of DnaK from *Mycobacterium tuberculosis,* human (mortalin, HSPA9), *Escherichia coli*, and *Geobacillus kaustophilus*. Residues in the interdomain linker sequence are highlighted in *blue*. SBD loop 1 residues are shown in *green* (*E. coli* K414–P419, *G. kaustophilus* R382–P388, *H. sapiens* mortalin HSPA9 R460–P465, and *M. tuberculosis* R388–P393). The residues that contact the GrpE coiled-coil domain are highlighted in *red*: Asn (*E. coli* N415, *G. kaustophilus* N383, mortalin HSPA9 N461, and *M. tuberculosis* N389), Thr (*E. coli* T417, *G. kaustophilus* T385, mortalin HSPA9 T463, and *M. tuberculosis* N391), and Asp (*E. coli* D477, *G. kaustophilus* D446, mortalin HSPA9 D523, and *M. tuberculosis* D451). *B,* sequence alignment of GrpE homologs where conserved residues are highlighted (hydrophobic in *green*, polar in *light blue*, negatively charged in *red*, positively charged in *blue*, and aromatic in *yellow*). The (1) L63–R75 region in the coiled-coil domain that interacts with DnaK is highlighted with a *red box*; the (2) I91–D110 region that contacts DnaK NBD subdomain IB; and the (3) L148–I157 region that interacts with DnaK NBD subdomain IIB are highlighted with *black boxes*. The numbering in this caption corresponds to the *E. coli* GrpE. *C,* sequence alignment for the N-terminal disordered tails of GrpE homologs. The only sequence similarity region in the N-terminal tails is highlighted (hydrophobic residues in *green*, negatively charged ones in *red*). DnaK, 70-kDa heat shock protein of prokaryote; GrpE, nucleotide exchange factor of prokaryote; NBD, nucleotide-binding domain; SBD, substrate-binding domain.

This comparison of the predicted *E. coli* DnaK–GrpE complex with structures of homologous complexes leads to the expectation that the interaction interfaces are conserved across species. An alignment of DnaK sequences from *E. coli*, *G. kaustophilus*, *Homo sapiens* mitochondrial, and *M. tuberculosis* reveals sequence identities ranging from 54% to 59% and similarities between 70% and 74%. Notably, the interdomain linker is highly conserved across all four variants, with nearly identical residue sequence and length (Fig. 2*A*). Loop 1 also maintains a conserved sequence, particularly the SBD N415 and T417 residues that interact with GrpE, which are preserved in all four species (Fig. 2*A*). In addition, the SBD D477 residue and the surrounding region exhibit sequence identity across the four Hsp70s analyzed (*E. coli* numbering) (Fig. 2*A*).

We explored the conservation of the structural features in the complexes with DnaK among different GrpEs. Representative GrpEs from different organisms were included in an alignment: GrpEL1 from *H. sapiens* mitochondria (eukaryotes), GrpE from *E. coli* and *Klebsiella pneumoniae* (gram-negative bacteria), and GrpE from *M. tuberculosis*, *G. kaustophilus*, and *Bacillus subtilis* (gram-positive bacteria). GrpE homologs from gram-negative species show exceptionally high overall sequence conservation, with 87% identity and 94% similarity, whereas the human and gram-positive counterparts exhibit lower overall identity and similarity percentages. However, all analyzed GrpEs display conserved stretches flanked by nonconserved regions (Figs. 1 and 2*B*). In particular, the residue sequence of the interaction interfaces between GrpE and DnaK NBD is highly conserved: residues I91–D110 in the β-bundles, which interact with DnaK subdomain IB, and the stretch L148–I157 in the α-helices, which interacts with DnaK subdomain IIB (*E. coli* numbering) (Fig. 2*B*). Notably, G122, which, when mutated to an Asp residue, disrupts the interaction between DnaK NBD and GrpE, and renders the NEF inactive (17, 24), is conserved across most variants. The motif interacting with DnaK SBD in the coiled-coil domain (residues L63–R75, *E. coli* numbering) also shows high sequence identity.

Given the proposed role of the N-terminal disordered tails of the *E. coli* GrpE in modulating the affinity of DnaK for a model peptide (5, 6, 7, 8), we examined sequence similarities for this region as well in the alignments (Fig. 2*C*). We were specifically seeking any likely regions within the tails that had features suggesting they could bind to the Hsp70 substrate-binding cleft (25). The N-terminal disordered tails of GrpE exhibit minimal sequence similarity among the set of structurally described homologs. To explore any potential conservation that did not emerge in this small set of sequences, we included additional prokaryotic homologs in our alignment, and two key features were observed: a high proportion of negatively charged residues, primarily Glu, and a conserved pattern of two or three hydrophobic residues flanked by negatively charged amino acids, typically Glu. In *E. coli*, these hydrophobic residues correspond to I17, I18, and M19.

### Binding of GrpE reduces the substrate-binding affinity of nucleotide-free *E. coli* DnaK, but binding of GrpE lacking its N-terminal 33-amino acids does not

**Figure 3:**
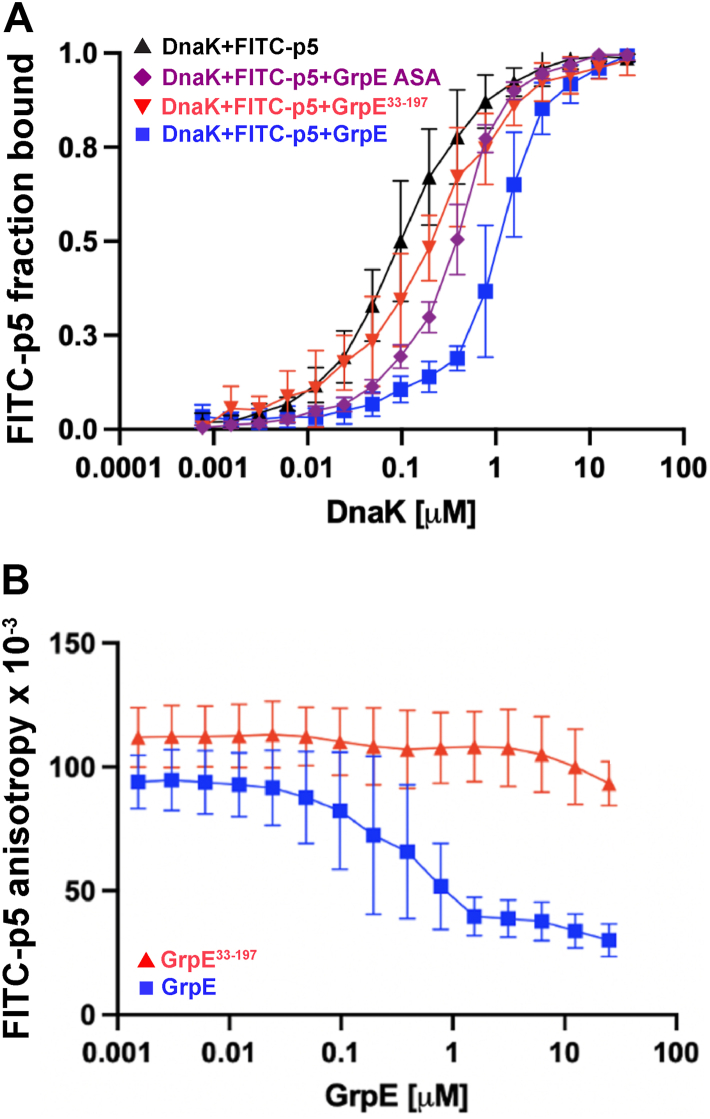
**The binding of GrpE reduces the affinity of the DnaK SBD for a model peptide**. *A,* apparent affinity of DnaK for the model peptide FITC-p5 in the absence or presence of 1 μM GrpE, GrpE^33–197^, and GrpE ASA at 22 °C. *B,* apparent affinity of DnaK for the model peptide FITC-p5 when the concentration of GrpE and GrpE^33–197^ was varied at 22 °C. DnaK, 70-kDa heat shock protein of prokaryote; GrpE, nucleotide exchange factor of prokaryote; SBD, substrate-binding domain.

Previous studies reported that GrpE complex formation with DnaK influences the kinetics of peptide binding and release in the presence of ADP (5, 6, 7, 9). Given that the nucleotide-free state is the functionally critical state of the DnaK NBD complex with GrpE (17), we tested the impact of GrpE binding to apo-DnaK on substrate binding. In this experiment, the amount of DnaK relative to peptide and GrpE was varied. We found that the apparent affinity (app-*K*_*D*_) of DnaK for the FITC-labeled peptide p5 (ALLLSAPRR (26)) was reduced in the presence of GrpE (Table 1, Fig. 3*A*). Importantly, removal of the N-terminal disordered tails (in GrpE^33–197^) diminished this effect such that the observed substrate affinity shifted toward that of isolated DnaK. We noted that the binding curve for the complex of DnaK with GrpE^33–197^ shows a small difference in substrate affinity relative to free DnaK; excitingly, this may be a consequence of an allosteric influence of the binding of the nontail portion of GrpE, and this aspect of the interaction will be pursued in subsequent studies (17). Nonetheless, the calculated app-*K*_*D*_ for the DnaK complex with GrpE^33–197^ was the same as that of the free chaperone within experimental error (Fig. S6*A*). We also found that in the presence of increasing concentrations of NEF, GrpE^33–197^ is still unable to stimulate peptide release (Fig. 3*B*). Thus, our results using apo-DnaK support a role for the GrpE tails in modulating DnaK substrate affinity, as previously described in the case of ADP-bound DnaK (5, 6, 7, 9).

**Table 1:** Apparent *K*_*D*_ of DnaK for FITC-p5 in the absence or presence of GrpE and its variants

Complex	22 °C	38 °C
	Apparent *K*_*D*_ of FITC-p5 (μM)	Apparent *K*_*D*_ of FITC-p5 (μM)
DnaK–GrpE	1.11 ± 0.06	0.76 ± 0.04
DnaK–GrpE^33–197^	0.21 ± 0.07	0.45 ± 0.04
DnaK–GrpE ASA	0.36 ± 0.03	—
DnaK alone	0.10 ± 0.07	0.48 ± 0.03

### NMR chemical shifts observed for the DnaK complex with GrpE argue that an Ile residue occupies the substrate-binding groove of the SBD

**Figure 4:**
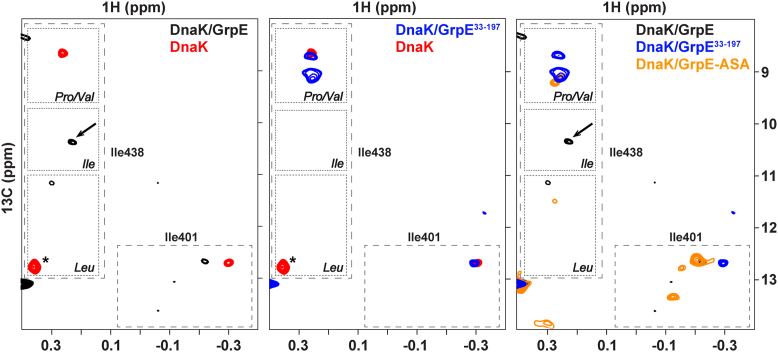
**DnaK SBD reporter NMR signals, I401 and I438, indicate which residues bind to the central pocket when complexed with GrpE and its variants**. Ile resonances of the substrate-binding pocket on the HMQC at 25 °C of (*left*) ^13^C-methyl-labeled ILV-DnaK–GrpE complex (*black*) and isolated apo-DnaK (*red*), (*middle*) ^13^C-methyl-labeled ILV-DnaK–GrpE^33–197^ complex (*blue*) and isolated apo-DnaK (*red*), and (*right)*^13^C-methyl-labeled ILV-DnaK–GrpE complex (*black*), ^13^C-methyl-labeled ILV-DnaK–GrpE^33–197^ complex (*blue*), and ^13^C-methyl-labeled ILV-DnaK–GrpE ASA complex (*orange*). An *arrow* marks the I438 peak in the ILV-DnaK–GrpE complex spectrum. The residue marked with an *asterisk* in the apo-DnaK spectrum corresponds to residue I338. DnaK, 70-kDa heat shock protein of prokaryote; GrpE, nucleotide exchange factor of prokaryote; HMQC, heteronuclear multiple quantum correlation; SBD, substrate-binding domain.

To further investigate the impact of GrpE binding on the DnaK SBD, we collected heteronuclear multiple quantum correlation (HMQC) NMR spectra of nucleotide-free, ^13^C-methyl ILV-DnaK in complex with GrpE or GrpE^33–197^. When DnaK forms a complex with GrpE, there are many chemical shift changes relative to free DnaK, including small and localized changes associated with the SBD. By contrast, formation of the DnaK complex with GrpE^33–197^ results in fewer perturbations within the SBD (Fig. S2), supporting a role for the disordered N-terminal tails. We focused our NMR analysis of the GrpE complex on the δ1-methyl chemical shifts of Ile 438 of the DnaK SBD, as this residue has proven to be an extremely useful reporter of the nature of the residue bound to the canonical substrate-binding pocket of the SBD (27). In the DnaK–GrpE complex, I438 exhibited a chemical shift indicative of binding of an Ile residue to the central pocket of the substrate-binding cleft (Fig. 4, *left panel*) (27). This I438 peak position is only observed in the DnaK–GrpE complex and not in nucleotide-free DnaK or in DnaK in complex with GrpE^33–197^ (Fig. 4), supporting occupancy of the SBD binding cleft by the GrpE N-terminal disordered tails. GrpE has two Ile in its tail (sequence ^14^PEE**II**MDQH^22^), and both are conserved among the different GrpE homologs (Fig. 2*C*). It is not possible to speculate about which Ile in the N-terminal tail is bound to the SBD pocket; however, the broad and low intensity of the SBD I438 δ1-methyl resonance suggests that the binding affinity of the GrpE N-terminal tail for the SBD is low, fully consistent with the numerous negative charges in the region of the Ile residues that may be binding to the SBD, and these are disfavored in canonical DnaK-binding sites (25).

Interestingly, in the DnaK–GrpE^33–197^ complex, the position of the I438 resonance is consistent with a Val or Pro residue occupying the central pocket (Fig. 4, *middle panel*) (27). While we lack a definitive explanation for this observation, we offer two potential explanations: one possibility is that a region of GrpE other than the N-terminal tail is occupying the canonical substrate-binding site in this complex. This idea is supported by the fact that the Pro–Val peak in the spectrum of the DnaK–GrpE^33–197^ complex disappears upon addition of the p5 peptide (Fig. S3), suggesting that the Pro–Val peak seen in the DnaK–GrpE^33–197^ complex indeed arises from interactions at the canonical substrate-binding site. Alternatively, the binding of GrpE that lacks the N-terminal tails may induce a conformational change in the SBD that is reflected in the chemical shifts of I438. Since the DnaK–GrpE^33–197^ complex shows substrate-binding affinity similar to that of nucleotide-free DnaK, it is possible that the α-helical lid adopts a more closed conformation over the β-subdomain of the SBD in the complex with GrpE^33–197^. This conformational state may be characterized by a shift in the resonance of I438. Further studies will be required to distinguish between these or any other possibilities.

### NMR analysis supports binding of GrpE N-terminal tails to the canonical substrate-binding site in the DnaK SBD through the ^17^IIM^19^ motif

Our NMR analysis shows that upon GrpE binding, the Ile δ1-methyl chemical shift of DnaK I438 is consistent with an Ile residue occupying the central pocket. We thus hypothesized that the conserved ^17^IIM^19^ motif in the N-terminal tails binds to the canonical substrate-binding site in the DnaK SBD and that its binding competes with substrate binding, thereby facilitating substrate release. To test this model, we mutated I17, I18, and M19 to A17, S18, and A19; this GrpE variant is named GrpE ASA. The mutant sequence is less hydrophobic than the native IIM; thus, it is predicted to bind more weakly to the central binding pocket, which typically favors nonpolar residues (25).

If the ASA sequence ablates the tail interaction with the SBD as we predict, GrpE ASA is not expected to greatly modulate the affinity of nucleotide-free DnaK for the model peptide FITC-p5. Indeed, binding of GrpE ASA, like that of GrpE^33–197^, showed a minimal decrease in the affinity of DnaK for its substrate (Table 1, Figs. 3*A* and S6*A*), as compared with GrpE, indicating that ^17^IIM^19^ substitution for ASA residues renders GrpE almost as ineffective as removal of the N-terminal tails. These results are consistent with our hypothesis that the ^17^IIM^19^ motif in the N-terminal disordered tails of GrpE binds to the canonical substrate-binding cleft of DnaK SBD.

We also analyzed the I438 region in the NMR spectrum of ^13^C-methyl ILV-labeled apo-DnaK in complex with GrpE ASA (Fig. 4, *right panel*) (27). In this complex, the peak corresponding to an Ile residue in the central pocket of the SBD observed in the apo-DnaK–GrpE spectrum is absent, and a new peak emerged in the region assigned to I438 when a Pro–Val occupies the central substrate-binding site. This peak position is close to that seen in the DnaK–GrpE^33–197^ spectrum, suggesting that similar mechanisms may be responsible for these chemical shifts. Overall, the substitution of the conserved ^17^IIM^19^ motif by ASA strongly reduces the ability of GrpE to stimulate substrate dissociation from DnaK’s central pocket, likely because of the absence of the tail binding motif.

### GrpE interactions with DnaK lead to both conformational and dynamic changes in GrpE N-terminal tails

**Figure 5:**
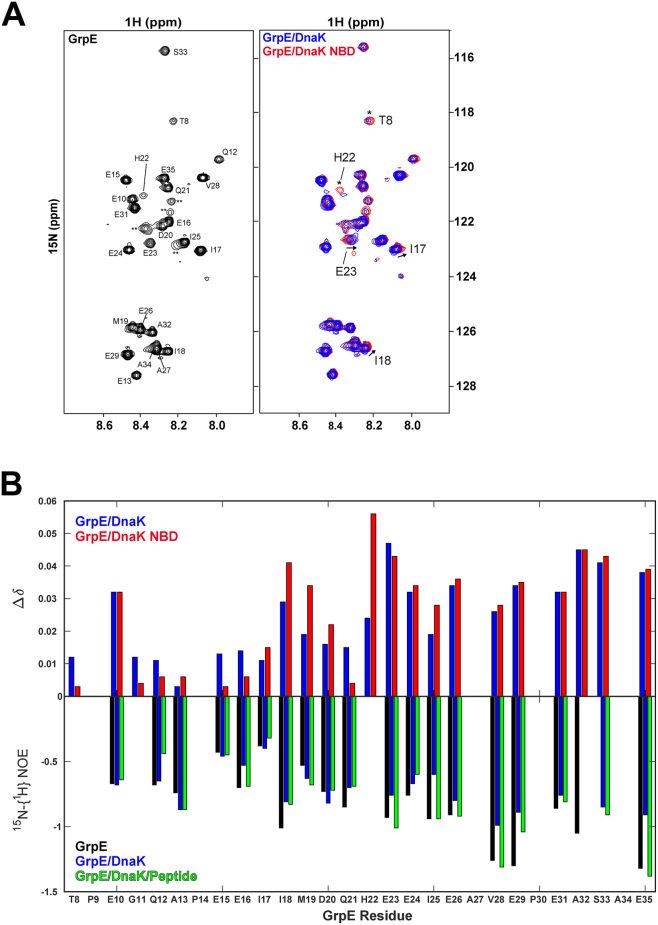
**^15^N-GrpE NMR analysis supports transient binding of the GrpE N-terminal tails to the DnaK SBD**. *A,* the ^1^H,^15^N-HSQC spectrum of ^15^N-GrpE (*left panel*) shows backbone amide resonances corresponding to disordered GrpE N-terminal tail residues. Resonance assignments are indicated, whereas unassigned peaks are labeled as ∗∗. An overlay (*right*) comparing equivalent spectra collected for the GrpE–DnaK complex (*blue*) and GrpE–DnaK–NBD (*red*) shows that the largest differences in peak position between the two complexes are observed for I17, I18, and E23 (indicated with *arrows*), whereas T8 and H22 experience more broadening in the GrpE–DnaK spectrum (indicated with *asterisks*). *B,* CSPs were measured for GrpE N-terminal amide signals in complex with DnaK (*blue*) or a truncated NBD (*red*) relative to those of isolated ^15^N-GrpE. *B, top,* bar plot comparing CSPs (Δδ) for ^15^N-GrpE in complex with full-length DnaK (*blue*) or the DnaK NBD^1–392^ (*red*) relative to ^15^N-GrpE alone. Missing CSPs correspond to proline residues (P9, P14, and P30) or shifts that could not be measured because of resonance overlap (A27, A34). *Bottom,*^15^N-{^1^H}-NOE values for GrpE (*black*), GrpE–DnaK (*blue*), and GrpE–DnaK–Ash2 peptide (*green*) complexes. Several NOEs are not indicated as there is a proline residue in the sequence (P9, P14, and P30), or there was resonance overlap resonance (A27 and A34), or there was insufficient intensity in the saturated spectrum (H22). CSP, chemical shift perturbation; DnaK, 70-kDa heat shock protein of prokaryote; GrpE, nucleotide exchange factor of prokaryote; HSQC, heteronuclear single quantum coherence; SBD, substrate-binding domain.

By observing ^15^N-labeled GrpE as it forms a complex with unlabeled DnaK, we were able to directly monitor chemical shift changes and alterations in the dynamics of the NEF. Due to the size and anisotropy of the GrpE, most of its NMR resonances are broadened and low intensity. However, the ^1^H–^15^N heteronuclear single quantum coherence spectrum of full-length ^15^N-labeled GrpE reveals around 30 distinct peaks (Fig. 5*A*), all of which are absent in the spectrum of ^15^N-labeled GrpE^33–197^ (Fig. S4), leading us to attribute these resonances to the N-terminal 33 residues. Assignment of this sequence, carried out following standard procedures (see the Experimental procedures section), confirmed that these resonances are from residues in the flexible N-terminal tails. Moreover, the observation of relatively narrow resonances for the N-terminal tails is fully consistent with the mobility of this region and the inability of either X-ray crystallography or cryo-EM methods to observe the N-terminal tails. For our study, the fact that this region is NMR observable is a highly informative finding.

A comparison of the chemical shifts of ^15^N GrpE free and in complex with DnaK shows perturbations in residues I17, I18, H22, and E23 of the GrpE N-terminal tails. Importantly, these CSPs are only present when full-length DnaK is used (Fig. 5*B*). These shifts are consistent with our model that the GrpE N-terminal tails interact transiently with the DnaK SBD. Importantly, the perturbations observed in I17 and I18 support the direct involvement of the ^17^IIM^19^ motif in this interaction. We propose that the CSPs of GrpE residues H22 and E23 upon complex formation arise because they are located toward the periphery of the substrate-binding pocket and are perturbed by contacts with the SBD (but not in the substrate-binding pocket).

If indeed the GrpE N-terminal tails are transiently associating with the SBD when the complex with DnaK is formed, as our model proposes, we expect that they would show reduced dynamic character. To probe the dynamics of this interaction, we performed ^15^N heteronuclear NOE experiments on ^15^N-GrpE, the ^15^N-GrpE–DnaK complex, and ^15^N-GrpE–DnaK in the presence of high concentrations of a high-affinity substrate peptide (Ash2: CRLLPLLFTPSR), which occupies the DnaK substrate-binding site and should outcompete the more weakly binding N-terminal tail (27). In free GrpE, the N-terminal residues exhibited negative NOE values, consistent with a disordered, dynamic region. Upon DnaK binding, there is a trend among residues following the ^17^IIM^19^ motif to show increased (less negative) NOE values, indicating reduced mobility, which is consistent with transient engagement of the N-terminal GrpE tails with the SBD (Fig. 5*B*). Notably, the region of the tail sequence that displays a trend toward reduced mobility based on ^15^N NOEs also shows a trend toward higher CSPs. Importantly, upon addition of a high-affinity DnaK binding peptide, Ash2, many of the residues from amino acids 15 to 33 showed their ^15^N NOE values shifting back to the levels seen in the free state. Taken together, these observations are consistent with displacement of the GrpE N-terminal tails from the SBD by the higher affinity peptide and thus add support for the model of transient binding of the GrpE tails *via* the ^17^IIM^19^ motif to the substrate-binding cleft of the SBD (Fig. 5*B*). Moreover, the transient binding of the GrpE tails is sensitive to substrate competition, highlighting a regulatory mechanism for substrate release.

### The impact of the GrpE N-terminal tails on peptide binding to the DnaK SBD is temperature dependent

*In vivo, E. coli* GrpE acts as a thermosensor for the DnaK allosteric cycle and modulates DnaK activity in response to temperature changes to maintain proteostasis (14, 15). Therefore, we tested whether the modulation of DnaK’s substrate affinity by GrpE is affected by temperature and how our proposed role for the GrpE N-terminal tails might inform the temperature-dependent function of GrpE.

**Figure 6:**
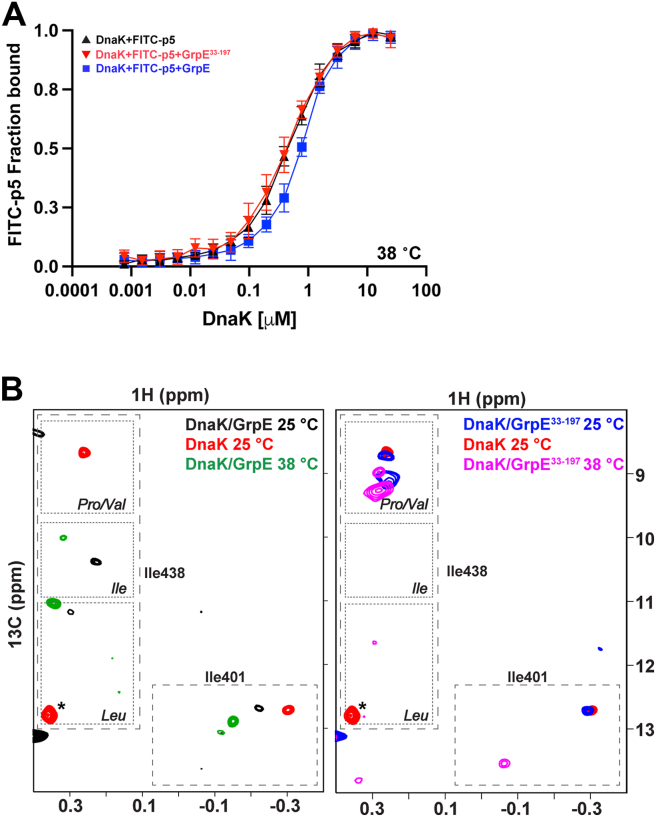
**GrpE modulates peptide release from apo-DnaK less at elevated temperatures**. *A,* apparent affinity of DnaK for the model peptide FITC-p5 in the absence or presence of 1 μM GrpE and GrpE^33–197^ at 38 °C. *B,* Ile resonances of the substrate binding pocket on the HMQC of (*left*) ^13^C-methyl-labeled ILV-DnaK–GrpE complex (*black*) at 25 °C, ^13^C-methyl-labeled ILV-DnaK–GrpE complex (*green*) at 38 °C, and isolated apo-DnaK (*red*) at 25 °C and (*right*) ^13^C-methyl-labeled ILV-DnaK–GrpE^33–197^ complex (*blue*) at 25 °C, ^13^C-methyl-labeled ILV-DnaK–GrpE^33–197^ complex (*pink*) at 38 °C, and isolated apo-DnaK (*red*) at 25 °C. The residue marked with an *asterisk* in the apo-DnaK spectrum corresponds to residue I338. DnaK, 70-kDa heat shock protein of prokaryote; GrpE, nucleotide exchange factor of prokaryote; HMQC, heteronuclear multiple quantum correlation.

We first determined DnaK’s apparent affinity for the FITC-p5 peptide (app *K*_*D*_) at 38 °C, a temperature reported to abrogate the NEF activity of GrpE (13, 16) in the absence or presence of GrpE or GrpE^33–197^. At 38 °C, the apparent affinity of DnaK for FITC-p5 is essentially unchanged upon addition of either GrpE or GrpE^33–197^, in contrast to the reduction of app-*K*_*D*_ of DnaK for this peptide upon binding GrpE at 22 °C (Table 1, Fig. 6*A* and S6*B*). To explain this finding, we hypothesize that the temperature-induced unraveling of GrpE’s coiled-coil domain (14) disfavors the transient interaction of the GrpE N-terminal tails with the substrate-binding pocket of DnaK SBD.

To test this hypothesis, we obtained the HMQC spectra of nucleotide-free ^13^C-methyl-labeled ILV-DnaK bound to GrpE or GrpE^33–197^ at 38 °C (Fig. 6*B*). At this temperature, the SBD I438 resonance in the DnaK–GrpE complex shifts from the region that corresponds to occupation of the substrate-binding pocket by an Ile residue, as observed at 25 °C. Interestingly, the SBD I438 resonance position for the DnaK–GrpE^33–197^ complex is at the same position regardless of the temperature (25 or 38 °C), and this position is compatible with a Pro or Val bound to the central pocket.

These experiments suggest that DnaK is able to bind the GrpE N-terminal tails at the temperature when GrpE retains its NEF activity and modulates substrate affinity, but when the temperature is raised such that the coiled-coil domain begins to unfold (13, 16), the interaction of the ^17^IIM^19^ motif in the GrpE N-terminal tails and the substrate-binding pocket is disfavored. Overall, these results help to explain why GrpE cannot assist DnaK refolding activity at higher temperatures.

## Discussion

Nucleotide exchange is pivotal to the physiological modulation of the allosteric cycle of Hsp70s. NEFs play a key role in this process, operating through a common mechanism. All NEFs bind to the ADP-bound NBD, inducing rotation and shifting of subdomain IIB to open the nucleotide-binding cleft and promote ADP release (4, 17). The release of ADP and subsequent binding of ATP trigger the transition of DnaK from the ADP-bound, high substrate affinity state to the ATP-bound, low substrate affinity state. Since NEFs promote the formation of the low substrate affinity state, it is not surprising that they have an influence on the affinity of DnaK for its substrate, as has now been reported by several laboratories (5, 6, 7, 8, 9).

While this previous research supports the role of NEFs in the dissociation of substrates from DnaK (and by extension other Hsp70s), the mechanism by which GrpE binding to the DnaK NBD affects substrate affinity was not clear. There has been support, largely based on studies of N-terminally truncated GrpE, that the N-terminal disordered tails of GrpE are key to its facilitation of peptide release (5, 6, 7, 8, 9), but no interaction of the tails and the SBD has been observed to date. In addition, many of these previous studies utilized ADP-bound DnaK, but we have recently shown that the ADP–DnaK–GrpE ternary complex is weak and populates an ensemble of interconverting conformations, with DnaK adopting a more stable GrpE complex in the absence of nucleotide (17).

Published structures of complexes between GrpE and DnaK of *E. coli* homologs have shed light on the nature of the interaction and how it might enable GrpE to modulate substrate affinity. Notably, the *M. tuberculosis* DnaK–GrpE complex (8), the *H. sapiens* mortalin–GrpEL1 complex (18), and the *G. kaustophilus* DnaK–GrpE complex (19), all revealed a conserved interaction interface between the DnaK SBD and the coiled-coil domain of GrpE, in addition to the previously identified interaction interfaces between the DnaK NBD and GrpE (7, 17). Furthermore, the structural ensemble of the *M. tuberculosis* DnaK–GrpE complex included structures in which the N-terminal tails were positioned closer to the SBD than observed in many other structures (8). We used the powerful new structure prediction method AlphaFold2 and found that the complex of *E. coli* DnaK and GrpE is very similar to that of the homologs. The conservation of key interaction regions between DnaK and GrpE suggests an evolutionary selection for function, indicating that these interactions are critical to the functionality of the DnaK–GrpE system. Our sequence alignments supported this conservation and thus the importance of the interaction interfaces. It is also reasonable to propose that the interaction of the DnaK NBD with GrpE’s globular domain acts as a driving force for the interaction between the SBD with the coiled-coil domain of GrpE.

We have made a number of observations, largely based on NMR, that taken together support a model in which formation of the DnaK–GrpE complex leads to transient binding of the GrpE N-terminal tails through their conserved ^17^IIM^19^ motif to the canonical substrate-binding site of the DnaK SBD. The important functional role played by weak, transient binding of the GrpE N-terminal tails to the DnaK SBDs is critically reliant on enhanced avidity from local concentration conferred by the formation of the complex between DnaK and GrpE. Consistent with this interpretation is the observation that a peptide consisting of residues 1 to 33 from GrpE’s N-terminal tails fails to compete with DnaK bound to the model peptide FITC-p5, even when titrated up to 200 μM (Fig. S5). Therefore, we conclude that the transient, weak binding of the GrpE N-terminal disordered tails to the DnaK SBD is key for *a functional* complex, as GrpE^33–197^ has reduced ability to stimulate peptide release and has been previously shown not to support DnaK assistance of luciferase refolding (5). The influence of the N-terminal disordered region on substrate binding in DnaK may also be enhanced by the allosteric impact of GrpE binding on the conformational landscape of the chaperone. While the allosteric impact of GrpE binding is indeed likely to favor the lower affinity of the SBD for its substrates, it nonetheless relies on the presence of the N-terminal disordered tails. Our proposed model involving direct, transient interaction of the N-terminal region explains the total set of observations, importantly including chemical shifts observed for the isoleucine δ1-methyl carbons in the SBD, which report reliably on occupancy of the substrate-binding cleft, and the model we propose can work in concert with the allosteric impact of GrpE binding.

The role of GrpE as a thermosensor is also elucidated by our studies of the temperature dependence of the N-terminal tail interaction with the DnaK SBD and its modulation of substrate binding to the DnaK SBD (14, 15). As the temperature increases, the coiled-coil domain of GrpE starts to unfold (13, 16), and GrpE loses its ability to assist DnaK in refolding substrates (9, 14, 15, 20). We conclude from our observations that this loss of GrpE coiled-coil domain stability is accompanied by a reduction of the ability to stimulate peptide dissociation, and moreover, that binding GrpE’s N-terminal tails to the DnaK SBDs is decreased because of the greater dynamics of a larger segment of the GrpE N-terminal region.

Overall, it is striking how nature has shaped the interaction between DnaK and GrpE to support efficient protein folding within the cell in a temperature-dependent manner. In addition, the work described provides a compelling example of a system in which structural properties of a complex can enhance the functional importance of a dynamic region of a protein by favoring weak, transient interactions that would otherwise be unlikely to be influential.

## Experimental procedures

### Protein expression and purification

Wildtype *E. coli* DnaK was expressed and purified as described previously (17, 28, 29). *E. coli* GrpE and GrpE^33–197^ were expressed in *E. coli* BL21(*DE3*), and GrpE ASA was expressed in *E. coli* C43(*DE3*) at 37 °C and purified as described previously (17). GrpE concentrations throughout the article always refer to the dimer (17).

To prepare ^13^C methyl-labeled ILV nucleotide-free DnaK and ^13^C methyl-labeled ILV nucleotide-free NBD^1–392^, established protocols were followed (30, 31, 32, 33).

### Sequence alignments

For the DnaK alignments, the following DnaK sequences were used: *M. tuberculosis* DnaK (UniProt ID: P9WMJ9), *E. coli* DnaK (UniProt ID: P0A6Y8), *H. sapiens* mortalin HSPA9 (UniProt ID: P38646), and *G. kaustophilus* DnaK (UniProt ID: Q5KWZ7). For the GrpE alignments, the following GrpE sequences were used: *M. tuberculosis* GrpE (UniProt ID: P9WMT4), *Legionella pneumoniae* GrpE (UniProt ID: 032481), *Staphylococcus aureus* GrpE (UniProt ID: P63191), *K. pneumoniae* GrpE (UniProt ID: B5XVJ9), *Enterobacter cloacae* GrpE (UniProt ID: A0A6S5K1M6), *Citrobacter amalonaticus* GrpE (UniProt ID: AOA24RQH2), *E. coli* GrpE (UniProt ID: P09372), *Salmonella enteritidis* GrpE (UniProt ID: B5QUG9), *Clostridioides difficile* GrpE (UniProt ID: Q182F1), *G. kaustophilus* GrpE (UniProt ID: Q5KWZ6), and *B. subtilis* GrpE (UniProt ID: P15874). Sequence alignments were created using the Clustal Omega (34) and BLAST (35, 36) algorithms.

### NMR spectroscopy and chemical-shift analysis

Methyl ILV ^13^C-HMQC spectra were obtained at 25 or 38 °C on a Bruker Avance III 600 MHz instrument and analyzed as described previously (17, 31). ^1^H,^15^N-heteronuclear single quantum coherence spectra for GrpE, GrpE^33–197^, GrpE–DnaK, GrpE–NBD, and GrpE–DnaK–peptide were acquired at 25 °C using a 600 MHz Bruker Avance Neo spectrometer. CSPs were calculated for the GrpE–DnaK and GrpE–NBD complexes using the following equation:

$$\Delta \delta =\sqrt{{\left(\delta_{H,complex}-\delta_{H,GrpE}\right)}^{2}+{0.14\left(\delta_{N,complex}-\delta_{N,GrpE}\right)}^{2}}$$

Heteronuclear NOEs were measured using an interleaved pulse sequence (Bruker pulse program hsqcoef3gpsi) and calculated as the ratio of saturated over nonsaturated peak intensity. Resonance assignments for the N-terminal amide signals were obtained using a combination of HNCACB, HNCO, and HN(CA)CO triple-resonance experiments, collected with a 700 MHz Bruker Avance Neo spectrometer.

### Substrate binding

The apparent affinities of DnaK for FITC-labeled peptide p5 (ALLLSAPRR) in the absence or presence of GrpE, GrpE^33–197^, or GrpE ASA were measured at 22 or 38 °C in a Biotek Synergy 2 micro plate reader (Biotek), with excitation at 485 nm and emission at 516 nm. DnaK (0–25 μM) was added to 35 nM FITC-p5 in HMK buffer (20 mM Hepes pH 7.6, 10 mM MgCl_2_, 100 mM KCl, and 1 mM DTT) in triplicate in the absence or presence of 1 μM GrpE, GrpE^33–197^, or GrpE ASA. The mixture was then incubated in 384-well plates for 4 h at 22 or 38 °C, after which the fluorescence anisotropy was measured. The data were analyzed as described previously (27). Statistical analysis was performed using a one-way ANOVA to determine the significance of differences between the GrpE variants. The same process was repeated with varying concentrations of GrpE. Here, 0 to 25 μM GrpE or GrpE^33–197^ was added to 35 nM FITC-p5 in HMK buffer with 1 mM DTT in triplicate in the presence of 0.5 μM DnaK. The same process was also repeated with varying concentrations of a peptide consisting of residues 1 to 33 from GrpE’s N-terminal tails. Here, 0 to 200 μM GrpE^1–33^ was added to 35 nM FITC-p5 in HMK buffer with 1 mM DTT in triplicate in the presence of 0.5 μM DnaK.

## Data availability

All data are included in this article or in the supporting information, except for the structure coordinates predicted by AlphaFold2, which are being deposited in ModelArchive. Resonance assignments for the *E. coli* GrpE disordered tails have been deposited in the Biological Magnetic Resonance Bank. The latter information can be shared upon request to Akshitha Maqtedar (amaqtedar@umass.edu) or Lila M. Gierasch (gierasch@biochem.umass.edu).

## Supporting information

This article contains supporting information.

## Conflict of interest

The authors declare that they have no conflicts of interest with the contents of this article.
