# Supplementary material for: The nucleotide exchange factor, GrpE, modulates substrate affinity by interaction of its N-terminal tails with the DnaK substrate–binding domain
Source: J Biol Chem. 2026 Jan 20;302(3):111176. doi: 10.1016/j.jbc.2026.111176 (PMC12915176; doi:10.1016/j.jbc.2026.111176)
Supplement: Supplementary Material [file mmc1.pdf]

Supporting information

The nucleotide exchange factor, GrpE, modulates substrate affinity by interaction of its N-terminal tails with the DnaK substrate-binding domain.

Akshitha Maqtedar[a*], Maria-Agustina Rossi[a*], Eugenia M. Clerico[a], Robert V. Williams[a], and Lila M. Gierasch[a,b]

Affiliations: [a]Department of Biochemistry & Molecular Biology and [b]Department of Chemistry, University of Massachusetts Amherst, Amherst, MA, 01003, USA.

Contains

Supporting Figures S1 – S6 with corresponding legends

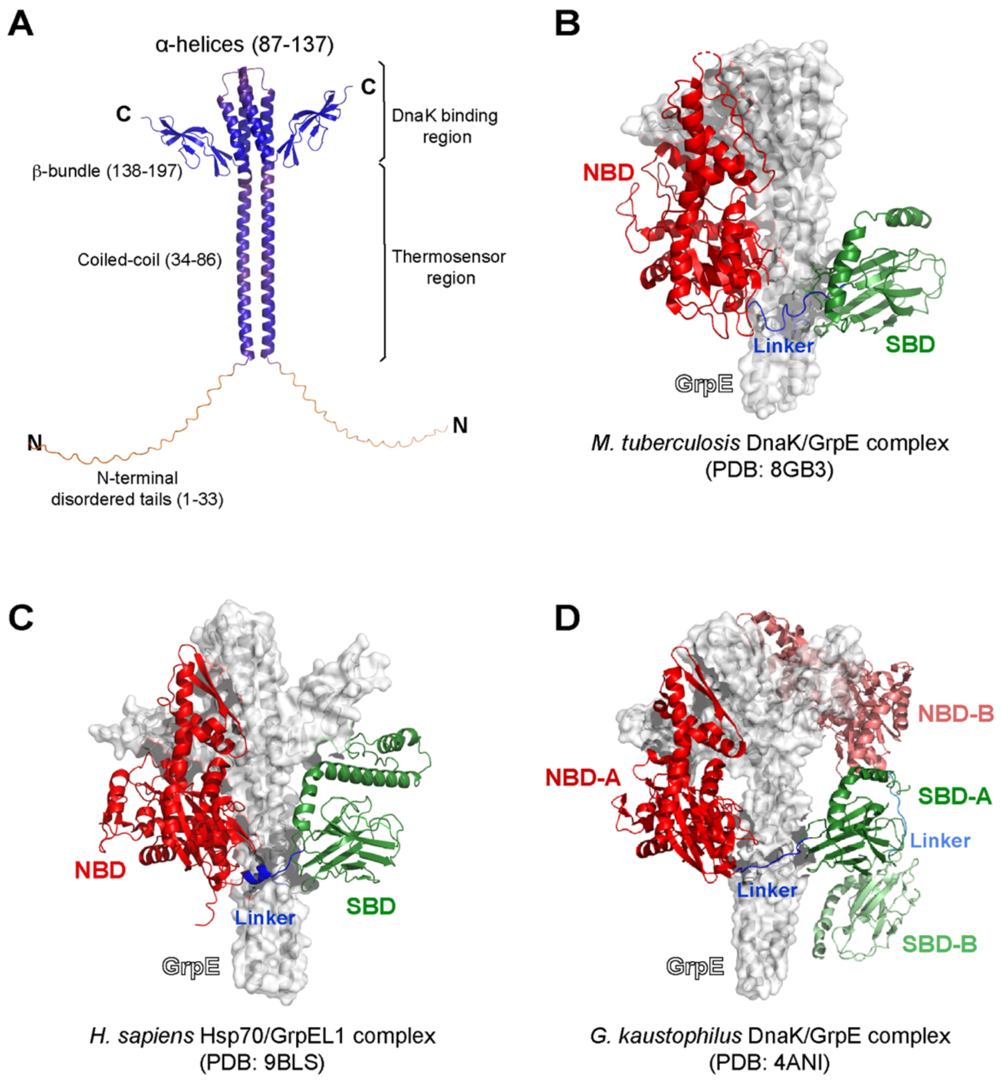

Figure S1. Structure of *E. coli* GrpE and structures of DnaK/GrpE complexes from *E. coli* homologues.

A, AlphaFold2-calculated structure of *E. coli* GrpE where the structural regions are indicated and colored by AlphaFold confidence (red = low confidence, blue = high confidence). *B-D*, Structures of diverse complexes between Hsp70s and GrpE homologs from various species. GrpE is always highlighted in white, and the Hsp70 NBD is in red, the SBD in green, and the interdomain linker in blue. *B,* CryoEM structure of the DnaK/GrpE complex from *Mycobacterium tuberculosis* (PDB: 8GB3); *C,* CryoEM structure of the mitochondrial GrpE/Hsp70 complex from *Homo sapiens* (GrpEL1 and mortalin; PDB: 9BLS); *D,* crystal structure of the DnaK/GrpE complex from *Geobacillus kaustophilus* (PDB: 4ANI), in which we show two DnaKs bound to GrpE (DnaK-A follows the standard coloring described above, whereas DnaK-B is colored as follows: NBD-B: salmon; SBD-B: light green; interdomain linker: sky blue).

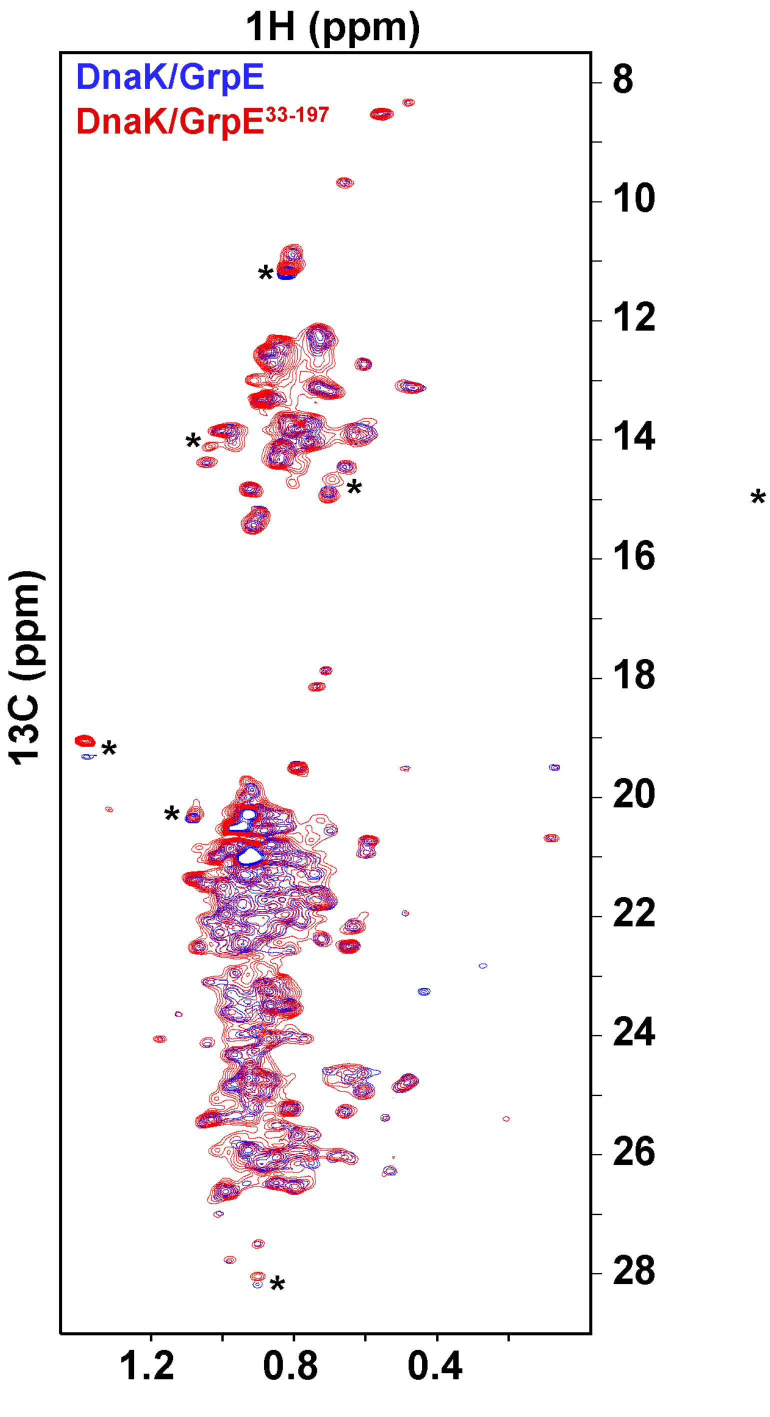

Figure S2. NMR chemical shift perturbations support binding of GrpE tail to DnaK SBD.

HMQC of ^{13}C-methyl labeled ILV-DnaK/GrpE complex (blue) and ^{13}C-methyl labeled ILV-DnaK/GrpE$^{33\text{-}197}$ complex (red) at 25 °C. Asterisks show residue examples with clear chemical shift perturbations.

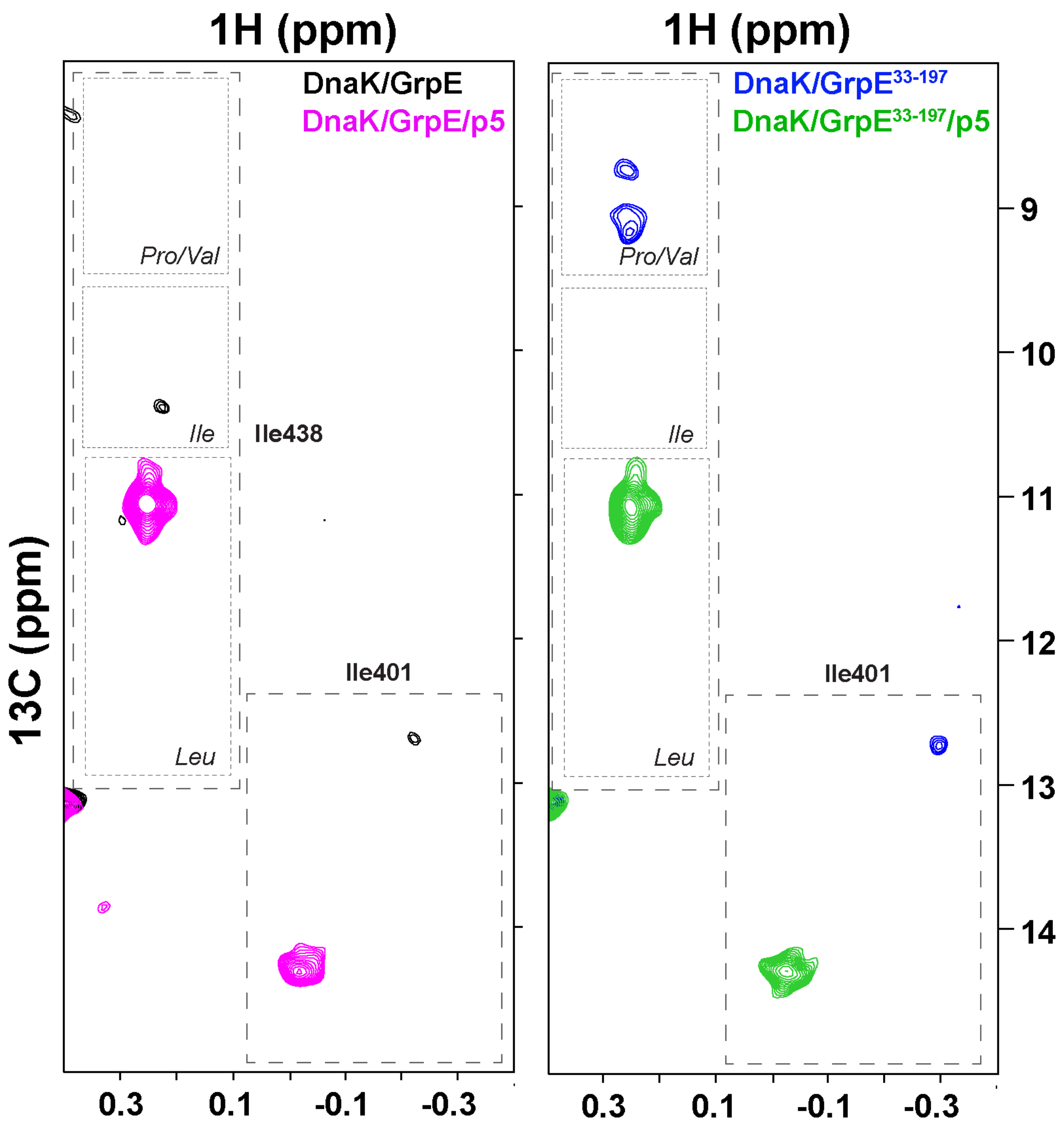

Figure S3. DnaK SBD reporter methyl NMR signals from residues I401 and I438 indicate the identity of residues binding to the central pocket of the SBD binding groove upon complex formation with GrpE and its variants and when the substrate p5 is added.

Ile resonances of the substrate binding pocket on the HMQC at 25 °C of (Left) ^{13}C-methyl labeled ILV-DnaK/GrpE complex (black) and ^{13}C-methyl labeled ILV-DnaK/GrpE/p5 complex (pink) and (Right) ^{13}C-methyl labeled ILV-DnaK/GrpE$^{33\text{-}197}$ complex (blue) and ILV-DnaK/GrpE$^{33\text{-}197}$/ p5 complex (green).

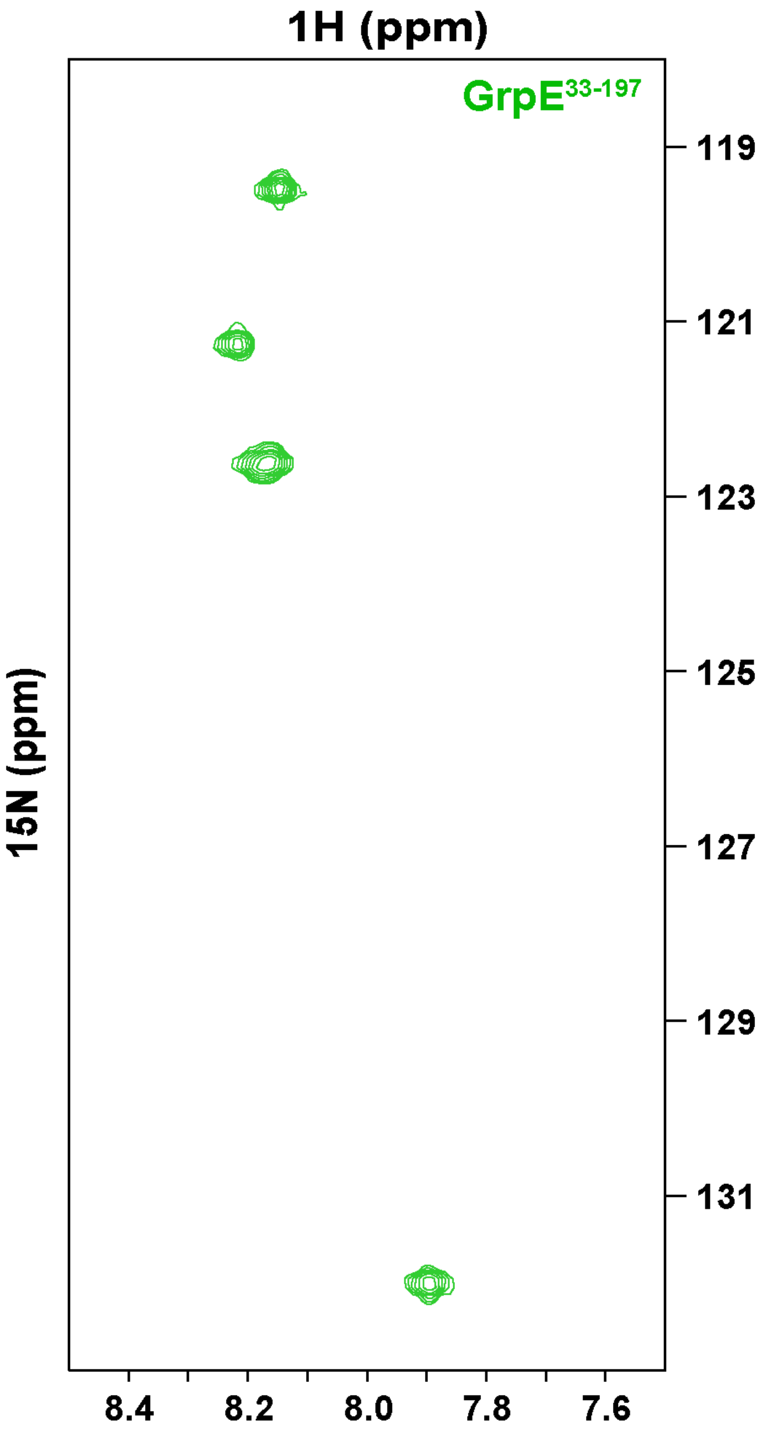

Figure S4. ^{15}N-GrpE$^{33\text{-}197}$ NMR experiments show that some remaining GrpE residues are dynamic.

^{1}H-^{15}N HSQC spectrum of the ^{15}N-labeled GrpE$^{33\text{-}197}$ spectrum (green).

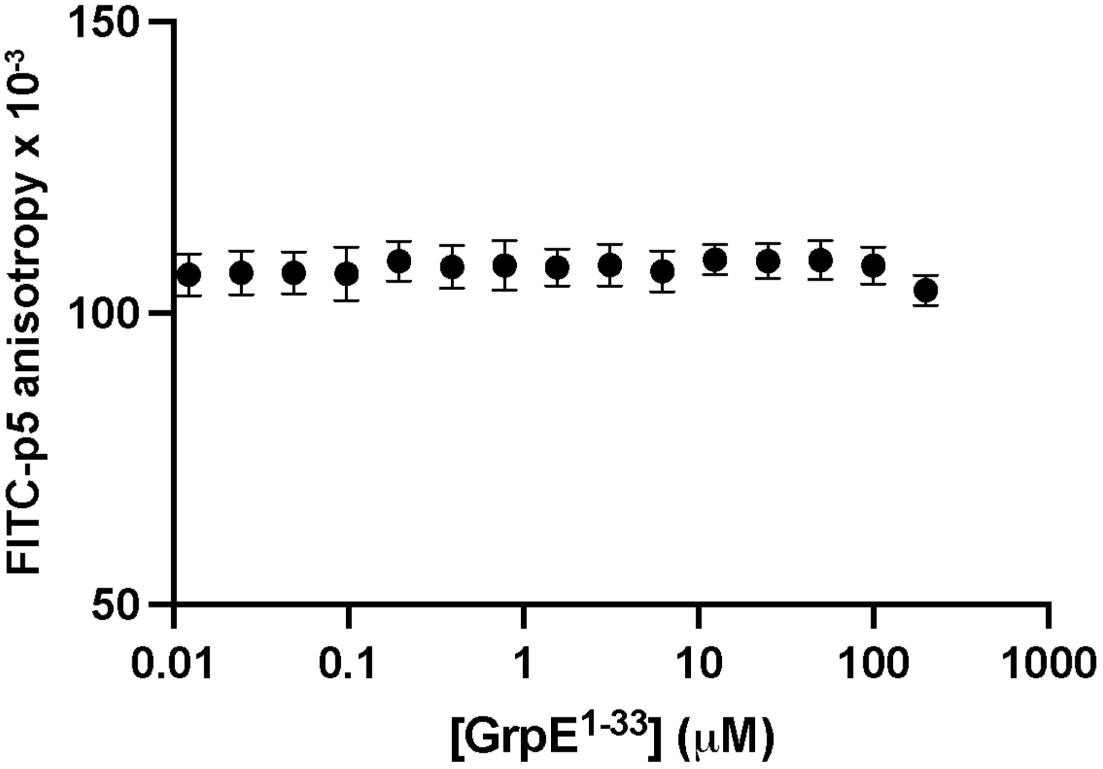

Figure S5. Peptide consisting of residues 1-33 of GrpE's N-terminal tails fails to compete with the model peptide FITC-p5 for DnaK binding.

Apparent affinity of DnaK for the model peptide FITC-p5 in the presence of 0-200 μM of a competing peptide consisting of residues 1-33 from GrpE N-terminal tails at 22 °C.

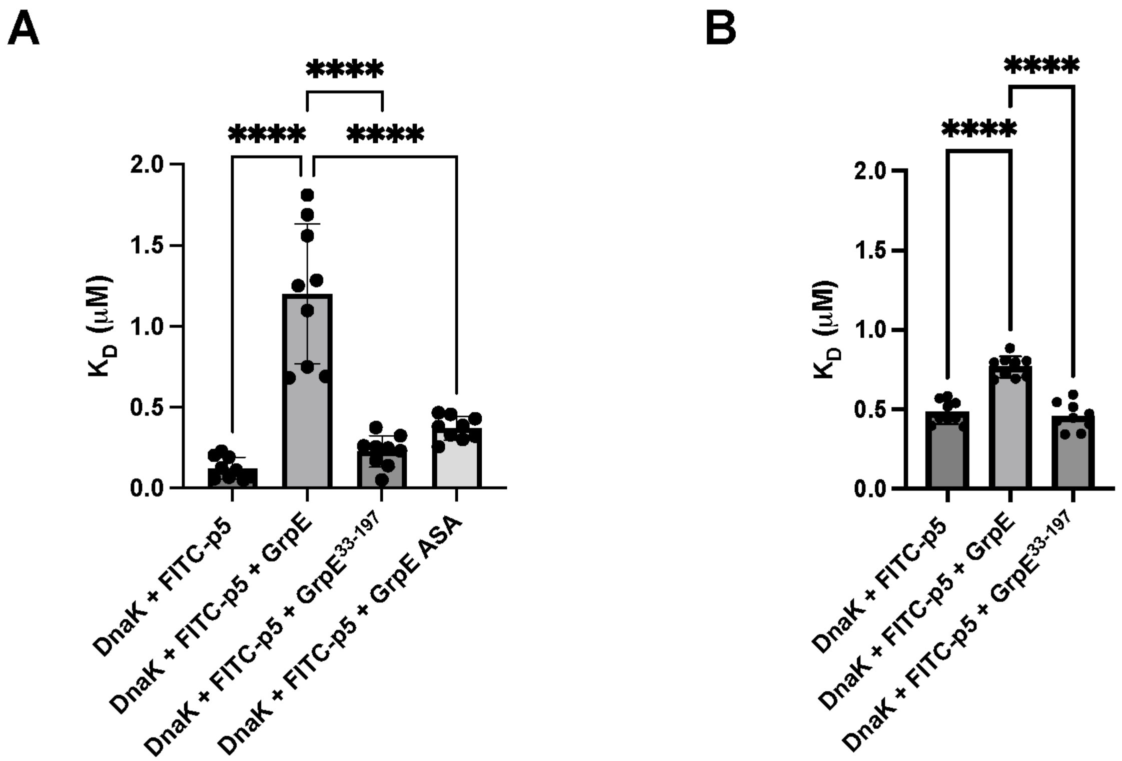

Figure S6. Statistical analyses of the apparent K_D of DnaK binding to the peptide FITC-p5 in the absence or presence of GrpE and its variants.

A, One-way ANOVA analysis for the apparent K_D's of DnaK for the model peptide FITC-p5 in the absence or presence of 1 μM GrpE, GrpE$^{33\text{-}197}$, and GrpE ASA at 22 °C. Significant differences were observed between DnaK + FITC-p5 vs. DnaK + FITC-p5 + GrpE ($p < 0.0001$), DnaK + FITC-p5 + GrpE vs. DnaK + FITC-p5 + GrpE$^{33–197}$ ($p < 0.0001$), and DnaK + FITC-p5 + GrpE vs. DnaK + FITC-p5 + GrpE ASA ($p < 0.0001$). No significant difference was observed for DnaK + FITC-p5 vs. DnaK + FITC-p5 + GrpE$^{33\text{-}197}$ ($p = 0.7574$), DnaK + FITC-p5 vs. DnaK + FITC-p5 + GrpE ASA ($p = 0.1167$), and DnaK + FITC-p5 + GrpE$^{33\text{-}197}$ vs. DnaK + FITC-p5 + GrpE ASA ($p = 0.5555$) *B,* One-way ANOVA for the apparent affinity of DnaK for the model peptide FITC-p5 in the absence or presence of 1 μM GrpE and GrpE$^{33\text{-}197}$ at 38 °C. Significant differences were observed between DnaK + FITC-p5 vs. DnaK + FITC-p5 + GrpE ($p < 0.0001$) and DnaK + FITC-p5 + GrpE vs. DnaK + FITC-p5 + GrpE$^{33–197}$ ($p < 0.0001$). No significant difference was observed for DnaK + FITC-p5 vs. DnaK + FITC-p5 + GrpE$^{33\text{-}197}$ ($p = 0.7345$. For each condition, experiments were performed in triplicate across three independent runs, yielding 9 independently determined K_D values for statistical analysis and plotting. Tukey's multiple comparisons test was used as the post hoc testing method for all pairwise comparisons.
